# Res-NeuS: Deep Residuals and Neural Implicit Surface Learning for Multi-View Reconstruction

**DOI:** 10.3390/s24030881

**Published:** 2024-01-29

**Authors:** Wei Wang, Fengjiao Gao, Yongliang Shen

**Affiliations:** 1 Intelligent Manufacturing Institute, Heilongjiang Academy of Sciences, Harbin 150090, China; 2211869@s.hlju.edu.cn; 2 College of Electronic Engineering, Heilongjiang University, Harbin 150080, China; 1995005@hlju.edu.cn

**Keywords:** surface reconstruction, neural radiance field, rendering, ResNet-50, appearance embedding

## Abstract

Surface reconstruction using neural networks has proven effective in reconstructing dense 3D surfaces through image-based neural rendering. Nevertheless, current methods are challenging when dealing with the intricate details of large-scale scenes. The high-fidelity reconstruction performance of neural rendering is constrained by the view sparsity and structural complexity of such scenes. In this paper, we present Res-NeuS, a method combining ResNet-50 and neural surface rendering for dense 3D reconstruction. Specifically, we present appearance embeddings: ResNet-50 is used to extract the appearance depth features of an image to further capture more scene details. We interpolate points near the surface and optimize their weights for the accurate localization of 3D surfaces. We introduce photometric consistency and geometric constraints to optimize 3D surfaces and eliminate geometric ambiguity existing in current methods. Finally, we design a 3D geometry automatic sampling to filter out uninteresting areas and reconstruct complex surface details in a coarse-to-fine manner. Comprehensive experiments demonstrate Res-NeuS’s superior capability in the reconstruction of 3D surfaces in complex, large-scale scenes, and the harmful distance of the reconstructed 3D model is 0.4 times that of general neural rendering 3D reconstruction methods and 0.6 times that of traditional 3D reconstruction methods.

## 1. Introduction

The objective of 3D reconstruction is to extract accurate information regarding the geometric structure of a scene from multiple images observed from varying viewpoints. The geometric structure information of the scene can be applied to a virtual reality scene representation or creating complete organ models in the medical field. At the same time, multi-view-based 3D reconstruction technology can be used in applications such as the digital reconstruction of cultural relics [1], traffic accident analysis [2], and other building site reconstructions [3].

The traditional approach to multi-view 3D reconstruction involves combining Structure from Motion (SFM) [4] with Multi-view Stereo Matching (MVS) [5,6,7,8]. Although impressive reconstruction results have been achieved, due to the cumbersome steps involved, cumulative errors are inevitably introduced into the final reconstructed geometric structure information. Moreover, an inherent limitation of this traditional algorithm is its inability to handle sparse, blurred views, such as areas with large areas of uniform color, complex texture areas, or remote sensing scenes captured from afar.

The latest 3D reconstruction methods represent scene geometric structure information as neural implicit surfaces and use volume rendering to optimize the surface to reduce biases caused by traditional multi-view reconstruction methods because volume rendering has greater robustness compared to surface rendering. Compared to the impression performance of indoor datasets (DTUs [9]) or some outdoor small-scene datasets taken at close range (we list some data from BlendedMVS [10]), the bias generated by traditional methods is partly optimized. However, when using only color information obtained via volume rendering to optimize the surface structure of a scene, challenges remain, specifically processing data in extreme weather conditions (cloudy or foggy, dark or daytime) and remote sensing scene data with distant, sparse views.

To overcome these challenges and apply neural rendering techniques to the above situations, we present a novel solution, Res-NeuS, for the high-fidelity surface reconstruction of multi-view complex scenes. We used the Signed Distance Function (SDF) [11,12,13,14,15] network to locate the zero-level set of a 3D surface and forward-optimized the volume-rendering color network through image appearance embedding [16]. We also added surface rendering to improve the original single-rendering framework to make the rendering process approximately unbiased and reversely optimize the SDF network by reducing the disparity between the rendered color and the actual color. Next, to address the issue of geometric ambiguity in that optimizing the scene geometry uses only color information, our method integrates multi-view stereo matching to constrain the geometry. Furthermore, to efficiently utilize computing resources and view dependency [17], we designed a coarse sampling scheme for automatically filtering interesting point clouds.

In summary, our contributions encompass the following: (1) we theoretically analyzed the biases in volume rendering, (2) based on the theoretical analysis, we present appearance embedding to optimize the color function, (3) we combine surface rendering and volume rendering, making the rendering results close to unbiased, (4) we integrate a multi-view stereo matching mechanism to constrain the 3D geometric structure, and (5) we present a novel geometric coarse sampling strategy. Compared to previous research work, we have improved the 3D geometric blur problem and further enriched colors to optimize the 3D model while simplifying the 3D reconstruction process.

## 2. Related Work

### 2.1. Multi-View Surface Reconstruction

Multi-view surface reconstruction is a complex process. For multi-view reconstruction with missing parts, the multi-view clustering method [18,19] can be used to restore image information, and then a 3D reconstruction of the scene can be performed. The purpose of multi-view surface reconstruction is to recover the exact geometric surface of a 3D scene from a multi-view image [20]. We summarize the merits and limitations of the multi-view 3D reconstruction method according to different representations, as shown in Table 1. In the initial stages of image-based photogrammetry techniques, a volumetric occupancy grid was employed to depict the scene. This process involves visiting each cube, or voxel, and designating it as occupied when there is strict adherence to color constancy among the corresponding projected image pixels. However, the feasibility of this approach is limited by the assumption of photometric consistency because auto-exposure and non-Lambertian materials would cause color inconsistency.

Subsequent approaches commonly initiate with 3D point clouds derived from multi-view stereo techniques, followed by a dense surface reconstruction. However, reliance on point cloud quality often leads to missing or noisy surfaces because point clouds are usually sparse. Recently, learning-based approaches have argued for carrying out the point cloud formation process by training neural networks. These approaches improve the quality and density of point clouds by learning image features and constructing cost volumes. However, they are limited by the cost volume resolution and fail to recover the geometric details of complex scenes.

### 2.2. Surface Rendering and Volume Rendering

Surface rendering [12,21,22,23]: The rendered color depends on the predicted color from the point at which the ray intersects with the surface geometry. When propagating backward, the gradients exit only at the local regions near the intersection. Hence, surface-based reconstruction methods encounter challenges in reconstructing complex scenes marked by significant self-occlusion and abrupt depth changes. Additionally, such methods typically necessitate object masks for supervision.

Volume rendering [24,25,26,27]: This is an image-based rendering method that renders a 3D scalar field into a 2D image. This method projects rays along a 3D volume. For example, NeRF [28] renders images by integrating the color of the sampling points on each ray, a process which can handle scenes with abrupt depth changes and synthesize high-quality images. However, achieving high-fidelity surface extraction from learned implicit fields [29] poses a challenge. Density-based scene representations face limitations due to insufficient constraints on their level sets. Therefore, the problem with photogrammetric surfaces is more direct to surface reconstruction.

### 2.3. Neural Implicit Surface Reconstruction

The neural implicit field is a new approach to representing the geometry of scenes by training a neural network to fit an implicit function on reconstruction. The inputs to this function are 3D coordinates, and the outputs are the characteristic values of scenes, such as distance or color. Meanwhile, the implicit function can be regarded as an implicit representation of the 3D scene. Therefore, to define the scene representation of 3D surfaces accurately [11,24,30,31,32,33,34,35,36], implicit functions such as occupancy grids [23,37] or signed distance functions are favored over straightforward volume density fields.

NeuS [24] is a classical neural implicit surface reconstruction method which applies volume rendering [24,25,28,37,38,39] to learn implicit SDF representation. However, applying standard volume rendering directly to the density values of Signed Distance Functions (SDFs) can lead to significant geometrical bias in scenes. Because the pixel weight is not on or near the object’s surface when the volume density is maximum, NeuS constructs a new volume density function and weight function to satisfy the above bias. When the volume density is the same and the distance from the camera is different, the weighted pixel of the point should be different.

### 2.4. Improvements in and Drawbacks of Neural Implicit Surface Reconstruction

Numerous experiments have shown on NeuS that volume rendering based on SDF is very beneficial for surface restoration from 2D images, particularly for some indoor small-scene datasets. Nonetheless, achieving high-quality 3D surface reconstruction remains a challenging task, particularly in the context of outdoor and large-scale scenes characterized by low visibility because the sparsity of view features can cause serious geometric deformation or distortion. Furthermore, the biases of the volume rendering paradigm (such as sample bias and weight bias) are greatly amplified when applied to such scenes.

## 3. Background

Our work extends NeRF [28] and its derivative NeuS [24]. In this summary, we encapsulate the pertinent aspects of these methods. For a more in-depth understanding, we recommend referring to the original papers.

### 3.1. NeRF and NeuS Preliminaries

The surface S of the scene is represented as follows:

(1) $$S=\left\{p\in R^{3}|f(p)=0\right\}$$

where f(p) is the signed distance function that maps a spatial position $p\in R^{3}$, and f(p)=0 represents a point on the surface of the observed object. This function can be represented by a neural network. It is called an SDF network in NeuS and is associated with NeRF in NeuS to optimize the SDF network using NeRF’s loss function.

For a specific pixel and a camera position o, we present a ray emitted by a camera and passing through a pixel as p(t)=o+tv,t≥0, where v is the unit direction vector of the ray and t is the depth along the ray starting at o. The volume rendering formula of classical NeRF is

(2) $$C(o,v)=\int_{t_{n}}^{t_{f}}T(t)\sigma (p(t))c(p(t),v)dt$$

To accurately describe volume density, the volume density must be at a maximum at or near the surface (when f(x)=0, σ(x) also reaches the maximum value, where the view direction $x\in R^{3}$ points to a color value), so NeuS redefined the expression of the volume density $\phi_{s}(u)=se^{-su}/(1+e^{-su})$, where u=f(x), the volume density expression $\phi_{s}(f(x))$ is called the S-density, and the rendering formula is

(3) $$C(o,v)=\int_{t_{n}}^{t_{f}}T(t)\phi_{s}(f(p(t)))c(p(t),v)dt$$

Let $w(t)=T(t)\phi_{S}(f(p(t)))$, and the w(t) function must be satisfied when the volume density is the same and the distance from the camera is different; the point Pixel weights should be different, otherwise there will be ambiguity. Furthermore, the weight function is normalized because of the influence of T(t):

(4) $$w(t)=\frac{\phi_{S}(f(p(t)))}{\int_{0}^{+\infty }\phi_{S}(f(p(t)))du}$$

Let w(t)=T(t)ρ(t) and $T(t)=\exp(-\int_{0}^{t}\rho (u)du)$, Therefore, T(t) and ρ(t) are solved. Meanwhile, NeuS completes the perfect combination of NeRF and surface reconstruction.

### 3.2. View Dependent on Sparse Feature Bias

NeuS’s scene representation is a pair of Multi-layer Perceptrons (MLPs). The first MLP receives sparse 3D points and camera position information x, outputs the S-density and a feature vector, and sends the feature vector with the 2D viewing direction, d, to the second MLP and outputs the color. The architectural design guarantees that the output exhibits distinct colors when observed from various viewpoints, using color to constrain the geometry, but the underlying shape representation is only a function of position. Therefore, only the feature encoding corresponding to sparse 3D points is considered, and the interval length between sampling points is ignored (sampling bias). This leads to missing finer details in appearance encoding.

### 3.3. Color Weight Bias

In volume rendering, when a ray traverses a scene, direct optimization involves the color integral of the sampling points to compute the rendered color. It is noteworthy that for indoor simple geometry datasets like DTU, the maximum of the color weight is typically concentrated on or near the surface position. However, in the case of remote sensing scenes, the color integration occurs along the entire ray rather than just at the surface intersection point. This distinction becomes particularly pronounced in scenes characterized by low visibility, long-distance, sparse views, and complex geometric shapes. The maximum of the color weight tends to deviate from the signed distance function (SDF) and is 0. Consequently, this color weight bias inevitably undermines the geometric constraint capability.

We define $C_{S}$ as the color at the point where the ray intersects with the object’s surface, and $C_{V}$ as the color of the volume rendering, $t^{*}=argmin\left\{t|o+tv=p,p\in \partial \Omega ,t\in (0,\infty )\right\}$, where ∂Ω represents the geometric surface. For neural rendering, we often obtain the SDF value through one MLP network inference and obtain the color field through another MLP network, which can be expressed mathematically as

(5) $$sdf(p)=F_{\Theta }(p)$$

(6) $$c(o,v,t)=C_{\varphi }(o,v,t)$$

The volume-rendered color of the pixel is written in discrete form as

(7) $$C_{V}=\sum_{i=1}^{n}w(t_{i})c(t_{i})=\sum_{\begin{array}{c} i=1\\ i\neq j \end{array}}^{n}w(t_{i})c(t_{i})+w(t_{j})c(t^{*})+w(t_{j})(c(t_{j})-c(t^{*}))$$

We presume that the initial intersection point of the ray and the surface is denoted as $p(t^{*})$ with $sdf(t^{*})=0$; the surface color at $p(t^{*})$ along the direction v, i.e., the surface rendering color can be expressed as

(8) $$C_{S}=c(t^{*})$$

For compositing new views, our goal is to make the color of the composite view consistent with the target color, so

(9) $$\Delta C=C_{V}-C_{S}=\frac{(1-w(t_{j}))c(t^{*})-\varepsilon_{sample}-\varepsilon_{weight}}{w(t_{j})}$$

$p(t_{j})$ is the nearest sampling point $p(t^{*})$, $\varepsilon_{sample}$ represents the deviation caused by the sampling operation, and $\varepsilon_{weight}$ represents the deviation caused by volume rendering weighting.

### 3.4. Geometric Bias

In many neural-rendering pipelines, geometry is commonly constrained by color loss obtained from a single view in each iteration. However, this approach lacks consistency across different views in the geometric optimization direction, introducing inherent ambiguity. As the input views become sparser, this ambiguity intensifies, leading to inaccuracies in the reconstructed geometry. Addressing this inherent ambiguity becomes especially challenging in the context of large-scale scenes, where views are frequently sparse.

## 4. Method

**Figure 1:**
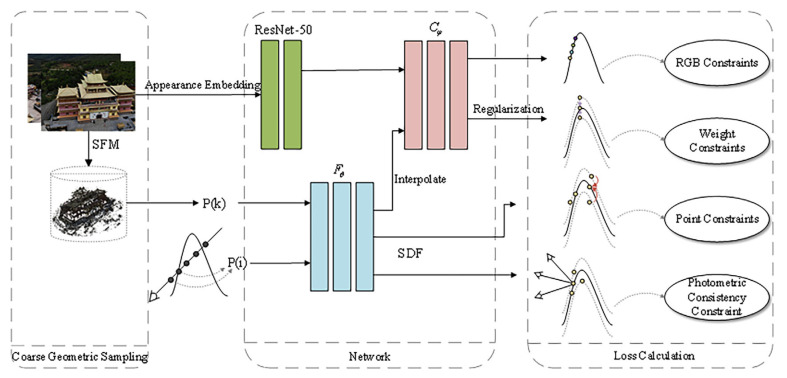
Overview of Res-NeuS. We incorporate ResNet-50 [40] into the network architectures of previous neural implicit surface learning methods. Subsequently, we interpolate the sampled points, estimate the color for all points, and optimize the color weights. Finally, we introduce the SDF loss derived from sparse 3D points and the photometric consistency loss from multi-view stereo to supervise the SDF network explicitly, additionally efficiently implementing coarse geometric sampling.

With a set of multi-view images and known poses at our disposal, our objective is to reconstruct surfaces that amalgamate the benefits of neural rendering and volume rendering, all without relying on mask supervision. We leverage the zero-level set of the signed distance function (SDF) to extract the scene’s surface in rendering to optimize the SDF. Firstly, we present a novel 3D geometric appearance constraint method known as image appearance embedding: this method involves extracting feature information directly from the images and feeding it into the color MLP, enhancing the disambiguation of geometric structures. Secondly, we perform interpolation on the sampling points of the volume rendering. Additionally, we apply weight regularization to eliminate color bias, as discussed in detail in Section 3.3, enhancing the overall rendering quality. Thirdly, we introduce display SDF optimization. This optimization is instrumental in achieving geometric consistency across the reconstructed scene, contributing to the overall accuracy of the 3D model. Lastly, we present an automatic geometric filtering approach aimed at refining the reconstructed surfaces. This method plays a crucial role in enhancing the precision and visual fidelity of the 3D model. Our approach overview is shown in Figure 1.

### 4.1. Appearance Embedding

**Figure 2:**
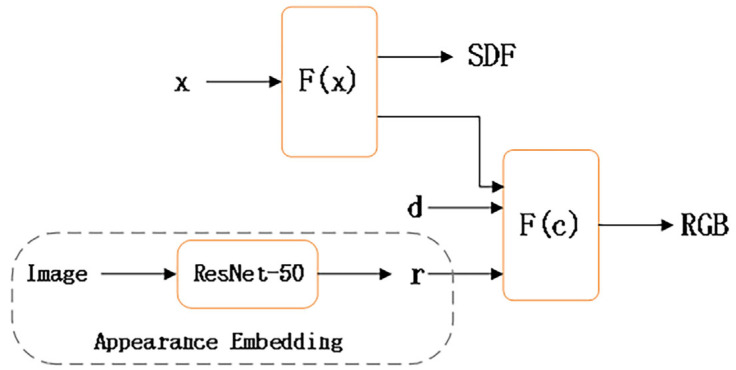
Integration of appearance embedding and neural implicit surface rendering.

To mitigate the sparse feature bias discussed in Section 3.2 and account for potential variations in environmental conditions during data capture [41], we extract appearance latent features from each image to subsequently optimize the color MLP. This process is illustrated in Figure 2.

In our model, the initial MLP is denoted as F(x), predicting the SDF for a spatial position x. Additionally, the network also generates a feature vector which is combined with the viewing direction d and an appearance embedding r. These amalgamated components are then fed into a second MLP denoted F(c) which produces the color corresponding to the given point. Therefore, the appearance embedding also further enriches the color information of the neural surface rendering, preparing for further accurate reconstruction.

During model training, considering that latent features typically diminish after repeated convolutions, ResNet-50 is employed to counteract this effect. Unlike conventional setups, ResNet-50 continuously incorporates previous latent features during the backward training process [40,42] thereby enhancing the global representation of features.

In addition, compared with ResNet-18 and ResNet-34, ResNet-50 not only improves the model’s accuracy but also significantly reduces the number of parameters and computations. The reason we did not choose ResNet-101 or ResNet-152 was because they require more computer memory. In the field of feature extraction, DenseNet [43] and MobileNet [44] have also produced impressive results. DenseNet directly merges feature maps from different layers to achieve feature reuse and improve efficiency, which is also the main difference from ResNets. However, the inherent disadvantage of DenseNet is that it consumes a lot of computer memory and cannot handle more complex images. In addition, the accuracy of MobileNet v3 large may decrease when dealing with complex scenarios, and the design of MobileNet v3 small is relatively simple, making it difficult to apply in complex scenarios. In summary, we chose ResNet-50 to extract the depth features of the image.

**Figure 3:**
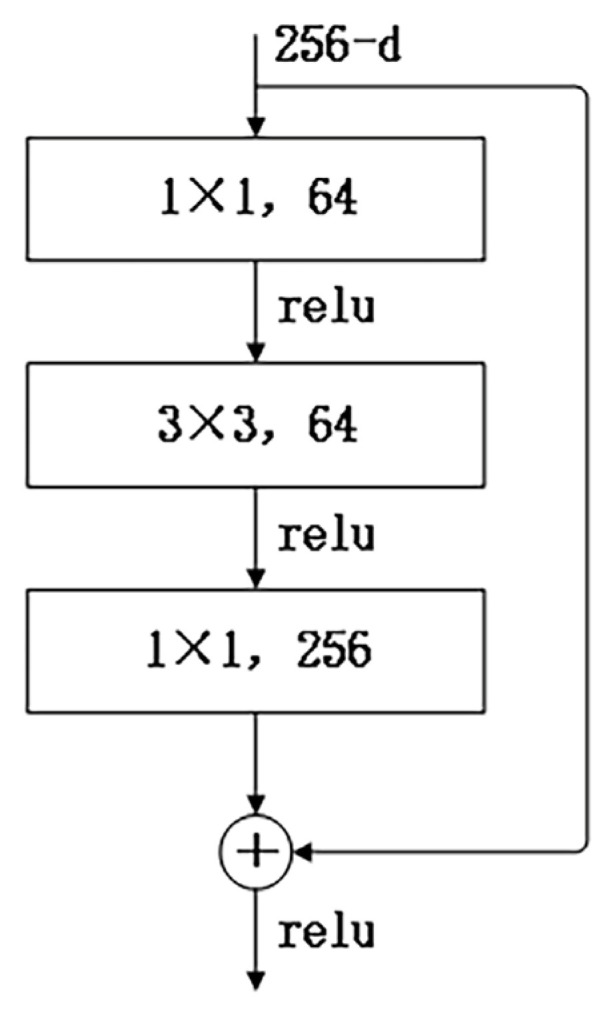
A building block “bottleneck” for ResNet-50.

Consequently, we crop the multi-view image of the scene to 224 × 224 and input the cropped image into ResNet-50 to extract useful features, and the output is a feature vector denoted as r=[1×1×256]. This vector is then fed into the color MLP to accomplish appearance embedding. The convolution results of each image input to ResNet-50, known as ImageNet are detailed in Table 2, and a bottleneck in ResNet-50 is illustrated in Figure 3.

ResNet-50 introduces a “Bottleneck” structure in the residual structure to reduce the number of parameters (multiple small-size convolutions replace a large-size convolution). This Bottleneck layer structure first goes through a 1 × 1 convolutional kernel, then a 3 × 3 convolutional kernel, and finally through another 1 × 1 convolutional kernel. The 256-dimensional input passes through a 1 × 1 × 64 convolutional layer, followed by a 3 × 3 × 64 convolutional layer, and finally through a 1 × 1 × 256 convolutional layer. Each convolutional layer undergoes ReLU activation, resulting in a total parameter count of 256 × 1 × 1 × 64 + 64 × 3 × 3 × 64 + 64 × 1 × 1 × 256 = 69,632.

**Figure 4:**
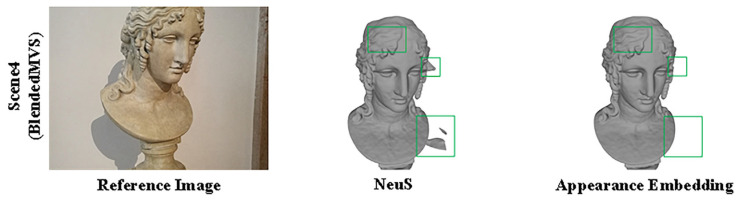
An illustration of the performance of NeuS and NeuS with appearance embedding on BlendedMVS. In comparison to NeuS, only embedding appearance demonstrates a substantial reduction in surface noise and a marked improvement in reconstruction accuracy.

**Figure 5:**
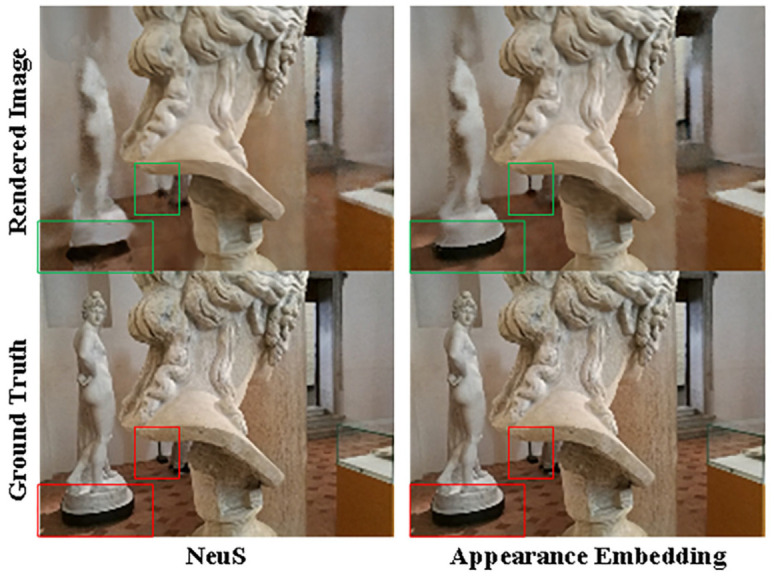
An illustration of rendering results; appearance embedding significantly enhances NeuS’s performance in view synthesis.

We assessed the surface reconstruction performance and view synthesis performance of NeuS and NeuS with embedded appearance features on the BlendedMVS dataset. As shown in Figure 4 and Figure 5 and Table 3 and Table 4. We assessed the performance of surface reconstruction using the distance metric. The chamfer distance is illustrated in Section 5.1.2. And the view synthesis performance was evaluated by PSNR/SSIM (higher is better) and LPIPS (lower is better) is illustrated in Section 5.1.2.

### 4.2. Volume Rendering Interpolation and Color Weight Regularization

To eliminate $\varepsilon_{sample}$ caused by the sampling operation mentioned in Section 3.3, first, identify two neighboring sampling points near the surface. Beginning at the camera position denoted as o,we move along the ray’s direction v, and their SDF values satisfy

(10) $$f(p(t_{i}))\cdot f(p(t_{i+1}))<0$$

The initial point of intersection between the ray and the surface, denoted as $P(t^{*})$, is approximated through linear interpolation as $p(\hat{t^{*}})$:

(11) $$p(\hat{t^{*}})=0+t^{*}$$

(12) $$t^{*}=\frac{f(p(t_{j}))t_{j+1}-f(p(t_{\left(j+1\right)}))t_{j}}{f(p(t_{j}))-f(p(t_{\left(j+1\right)}))}$$

Then, we incorporate the point set $p(\hat{t^{*}})$ into the initial point set $P(t_{i})$, resulting in a new point set $P=p(\hat{t^{*}})\cup P(t_{i})$. This combined set P is utilized to generate the final volume rendering color:

(13) $$C_{V-final}=\sum_{i=0}^{n}w(t_{i})c(t_{i})+w(\hat{t^{*}})c(\hat{t^{*}})$$

where $w(t^{*})$ represents the weight of $P(t_{i})$, $c(t_{i})$ represents the pixel value of $P(t_{i})$. $w(\hat{t^{*}})$ represents the weight of $p(\hat{t^{*}})$, $c(\hat{t^{*}})$ represents the pixel value of $P(t^{*})$, and n denotes the number of points. Then, the color bias becomes

(14) $$\begin{array}{l} \Delta C_{final}=\sum_{i=1}^{n}w(t_{i})c(t_{i})+w(\hat{t^{*}})c(\hat{t^{*}})-c(t^{*})=\sum_{i=1}^{n}w(t_{i})c(t_{i})+(w(\hat{t^{*}})-1)c(t^{*})\\ +c(\hat{t^{*}})-c(t^{*})=\varepsilon_{weight-final}+c(\hat{t^{*}})-c(t^{*})=\varepsilon_{weight-final}+\varepsilon_{interp} \end{array}$$

Following interpolation, we obtain $\varepsilon_{interp}$, signifying the bias introduced by linear interpolation. Importantly, $\varepsilon_{interp}$ is at least two orders of magnitude smaller than $\varepsilon_{sample}$.

Meanwhile, we also alleviate the weight bias to regularize the weight distribution:

(15) $$L_{weight}=\sum_{i=1}^{n}w(t_{i})|t_{i}-t^{*}|$$

$L_{weight}$ is utilized to eliminate anomalous weight distributions, specifically those located far from the surface yet exhibiting substantial weight values. This indirectly promotes the convergence of the weight distribution toward the surface. Theoretically, as the weight approaches $\delta (t-\hat{t^{*}})$, a delta distribution centered at $\hat{t^{*}}$, $\varepsilon_{weight-final}$ will tend towards 0.

### 4.3. Geometric Constraints

In the scenario of geometric ambiguity outlined in Section 3.4, we introduce photometric consistency loss and point constraints to illustrate the 3D representation of the supervised Signed Distance Function (SDF).

#### 4.3.1. Photometric Consistency Constraints

For a small area S on the surface, its small pixel patch on the projection of the source view is q. The patches associated with S are expected to exhibit geometric consistency across various source views except for occlusion instances. We use the camera coordinate of the reference image pixel $I_{r}$ to represent S, as follows:

(16) $$n^{T}p(\hat{t^{*}})+d=0$$

We introduce a homography matrix H to local the pixel value of a point $x_{i}$ in the reference image. And corresponding to the points x in other images, we have

(17) $$x_{i}=H_{i}x,H_{i}=K_{i}(R_{i}R_{r}^{T}-\frac{R_{i}(R_{i}^{T}t_{i}-R_{r}^{T}t_{r})n^{T}}{d})K_{r}^{-1}$$

where $K_{r}$ and $K_{i}$ are the internal calibration matrices, $R_{r}$ and $R_{i}$ are rotation matrices, $t_{i}$ and $t_{r}$ are translation vectors of the source view $I_{i}$ and other views $I_{r}$ respectively.

To measure the photometric consistency of different views, we introduce normalization cross-correlation between the reference image and source view

(18) $$NCC(X,H_{i}X)=\frac{Cov(X,H_{i}X)}{\sqrt{Var(X,H_{i}X)}}$$

where Cov denotes covariance and Var denotes variance, we use the rendered image as the reference image. We calculate Normalized Cross-Correlation (NCC) scores between the sampled patches and their corresponding patches in all source images. To address occlusions, we identify the top four computed NCC scores for each sampled patch [45] and leverage them to calculate the photometric consistency loss for the respective view:

(19) $$L_{photo}=\frac{\sum_{i=1}^{4}1-NCC(X,H_{i}X)}{4}$$

#### 4.3.2. Point Constraints

In the previous data-processing process, acquiring images with known camera poses was imperative. The position information of these images is estimated using Structure from Motion (SFM). SFM is also responsible for reconstructing sparse 3D points, and while these points unavoidably contain noise, they maintain a certain level of accuracy. Therefore, we represent these sparse 3D points $P_{0}$ to directly supervise f(P):

(20) $$L_{SDF}=\sum_{P\in P_{k}}\frac{1}{N}|f(P)|$$

where N represents the number of points contained within $P_{k}$. SFM reconstructs these points as $P_{k}$. We assume that any point P within $P_{k}$ is on the surface and its corresponding SDF value is denoted as f(P).

### 4.4. Point Cloud Coarse Sampling

In most scenarios, the majority of a scene is characterized by open space. In consideration of this, our objective is to strategically identify the broad 3D regions of interest before engaging in the reconstruction of intricate details and view-dependent effects, which typically demand substantial computational resources. This approach allows for a significant reduction in the volume of points queried along each ray during the subsequent fine-stage processing.

**Figure 6:**
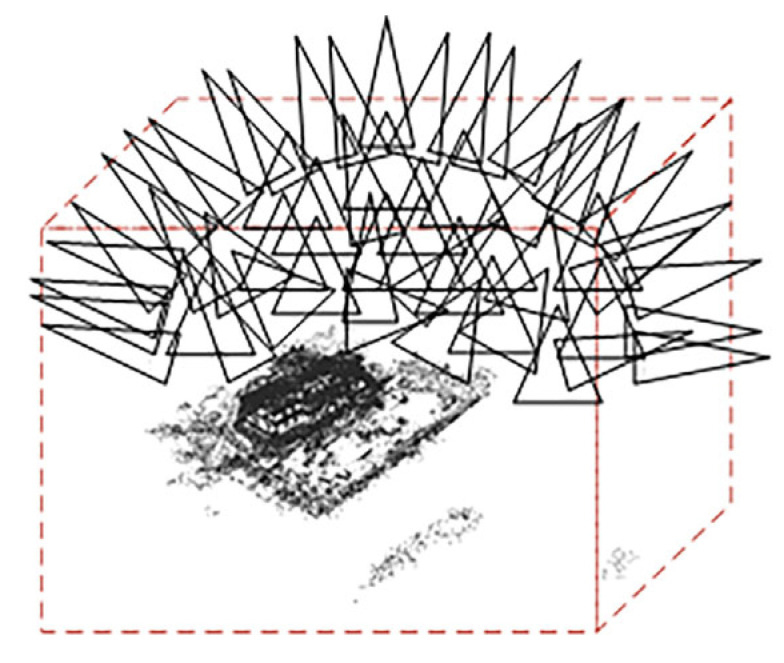
DVGO’s geometric coarse sampling.

In the handling of input datasets, conventional methods involve manual filtration to eliminate irrelevant point clouds. In contrast, DVGO [17] accomplishes the automatic selection of the point cloud of interest, representing a notable advancement in streamlining this process. To determine the bounding box, rays emitted by each camera intersect with the nearest and farthest points in the scene, as shown in Figure 6.

**Figure 7:**
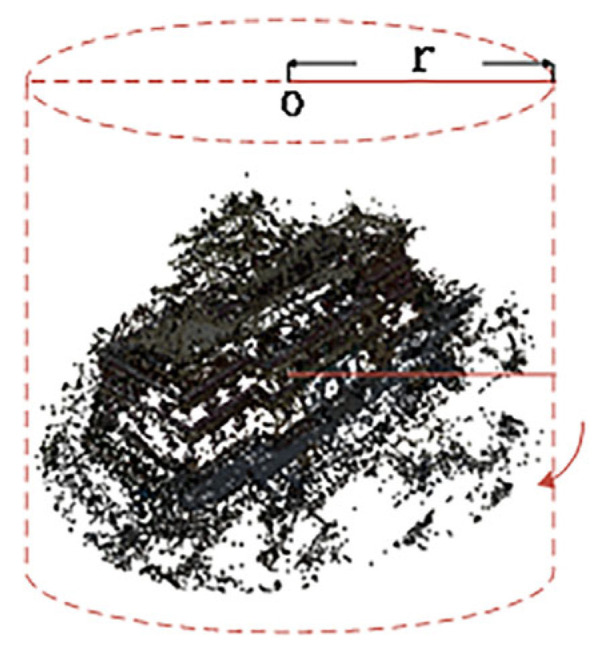
Res-NeuS’s geometric coarse sampling.

Due to the limitations and excessive size of the 3D point cloud regions selected by DVGO, precise localization of fine scene structures is not achieved. Therefore, we introduce a novel point cloud automatic filtering method. Leveraging camera pose information, we identify the point cloud center and compute the average distance from the center to the camera position. Using this average distance as the radius, we select a point cloud region of interest encompassing 360° around the center. The radius r defining the surrounding area is determined based on the camera’s capture mode, whether it is capturing a panoramic view or covering a distant scene, as shown in Figure 7.

### 4.5. Loss Function

The total loss is characterized as the weighted summation of individual losses:

(21) $$L=L_{\mathrm{RGB}}+\lambda L_{\mathrm{weight}}+\alpha L_{\mathrm{photo}}+\beta L_{\mathrm{SDF}}$$

## 5. Experiments

### 5.1. Exp Setting

#### 5.1.1. Dataset

We used the BlendedMVS dataset and the DTU dataset to verify the effectiveness of our method. This dataset encompasses scenes with a focus on large-scale scenes, as well as scenes featuring diverse categories of objects. The images in the dataset have a resolution of 768 × 576, and the number of views varies from 56 to 333. The evaluation of the reconstructed surfaces on the BlendedMVS dataset was conducted using chamfer distances in 3D space. Additionally, for the DTU dataset, we present the visual impact of the reconstructed surfaces.

#### 5.1.2. Evaluation Metrics

We assessed the performance of surface reconstruction using a distance metric. The chamfer distance in 3D space is mainly used for reconstruction work and is defined as follows:

(22) $$Acc=d_{CD}(S_{1},S_{2})=\frac{1}{S_{1}}\sum_{x\in S_{1}}\underset{y\in S_{2}}{min}||x-y|{|}_{2}^{2}$$

(23) $$Comp=d_{CD}(S_{2},S_{1})=\frac{1}{S_{2}}\sum_{y\in S_{2}}\underset{y\in S_{2}}{\min}||y-x|{|}_{2}^{2}$$

In the provided formula, $S_{1}$ denotes the ground truth sampling point, and $S_{2}$ represents the sampling point on the reconstructed surface. The evaluation metric for reconstruction accuracy (Acc) is defined as the chamfer distance from $S_{1}$ to $S_{2}$. Conversely, the evaluation metric for reconstruction completeness (Comp) is determined by the charmful distance from $S_{2}$ to $S_{1}$. The overall score is then computed as the mean of accuracy and completeness. A smaller distance implies a superior reconstruction effect.

Additionally, we assessed the performance of view synthesis akin to NeRF using image quality assessment metrics, including the Peak Signal-to-Noise Ratio (PSNR), Structural Similarity (SSIM), and Learned Perceptual Image Patch Similarity (LPIPS).

#### 5.1.3. Baselines

For a more comprehensive evaluation of our method, we conducted a comparative analysis by benchmarking it against the state-of-the-art learning-based method NeuS and the traditional multi-view reconstruction method COLMAP. This comparison is based on both the reconstruction effect and the evaluation indicators of the model.

#### 5.1.4. Implementation Details

Similar to [12], the SDF network and the color network were modeled by an eight-layer MLP and a four-layer MLP with 256 hidden units, respectively. Assuming that the target reconstruction area was confined within a sphere, we employed a batch size of 2048 rays during the sampling process. For each ray, we first sampled 32 points uniformly and then sampled 96 points hierarchically. The model was trained on a single NVIDIA GeForce RTX 4090 GPU, the learning rate was set to 5 × 10^−4^, and the training process spanned 50,000 iterations, taking approximately 4 h to fulfill memory constraints. After completing the network training, a mesh can be generated from the SDF within a predefined bounding box. This was achieved using the Marching Cubes algorithm [25] with a specified volume size of 512.

### 5.2. Experimental Results

**Figure 8:**
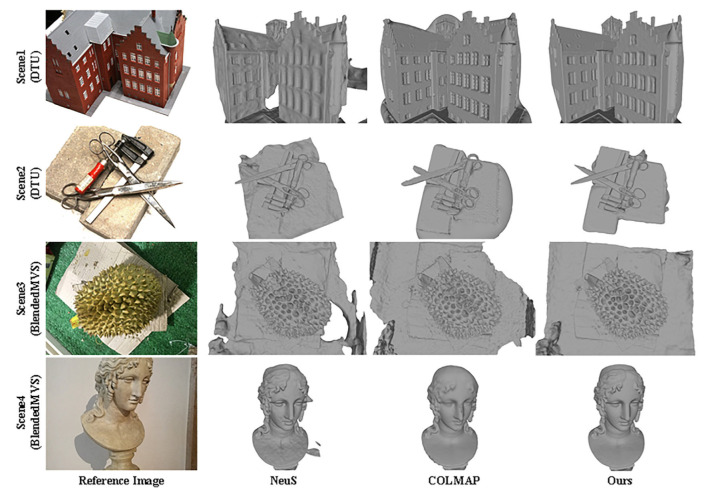
Qualitative surface reconstruction results for the DTU dataset and BlendedMVS dataset.

First, we used the reconstruction methods mentioned in Section 5.1.3 to test two indoor scenes in the DTU data set and two small scenes in the BlendedMVS data set and compared the reconstruction results; as shown in Figure 8, the test results show that our method is largely better than baselines.

**Figure 9:**
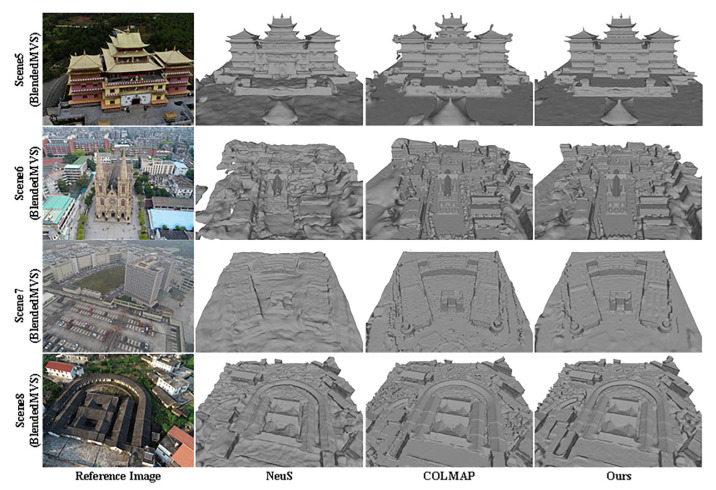
Qualitative surface reconstruction results on BlendedMVS.

Given the effectiveness of our method in reconstructing small scenes, we proceeded to apply the approach to larger scenes characterized by low visibility and sparse feature views typical of remote sensing scenes in the BlendedMVS dataset. The resulting reconstruction outcomes were compared and analyzed; qualitative surface reconstruction results are depicted in Figure 9 and quantitative surface reconstruction results are depicted in Table 5. Notably, the surfaces reconstructed by COLMAP exhibited noticeable noise, while NeuS, relying solely on color constraints, displayed severe deformations, distortions, and holes in the geometric surface structure. In contrast, our method excels in reconstructing accurate geometric structures while effectively eliminating smooth surface noise. For instance, it successfully reconstructs the geometry of scene 7 with low visibility and restores depth variations in scene 8.

We tested three methods using 14 challenging scenes from the Blended MVS dataset. The original picture of the scene is given in Appendix A. All three methods were performed without mask supervision, and the experimental setup of NeuS [24] was shown in the original paper. The details of the Res-NeuS implementation are shown in Section 5.1.4. We used the point cloud coarse sampling strategy mentioned in Section 4.4 to select the bounding box, which greatly saved the time of manually obtaining the bounding box, to facilitate the subsequent efficient reconstruction work. The bounding box applied to the different methods is the same for each scene processed. And the surface produced by COLMAP is trimmed with a trimming value of 0.

The quantitative results of the reconstruction integrity of COLMAP in scene 6 and scene 7 were better than our methods. But their visualizations are not very good; a reasonable explanation for this contradiction is that there were plenty of redundant surfaces located on the back of the visible surfaces in all cases, as shown in Figure 9. The redundant surfaces severely reduced the Comp value for scene 6 and scene 7. Except for scene 6 and scene 7, the visualization surface and Comp values of our method are better than those of NeuS and COLMAP. And the Comp value of our method is about 0.6 times that of COLMAP and 0.4 times that of NeuS.

### 5.3. Ablation Study

**Figure 10:**
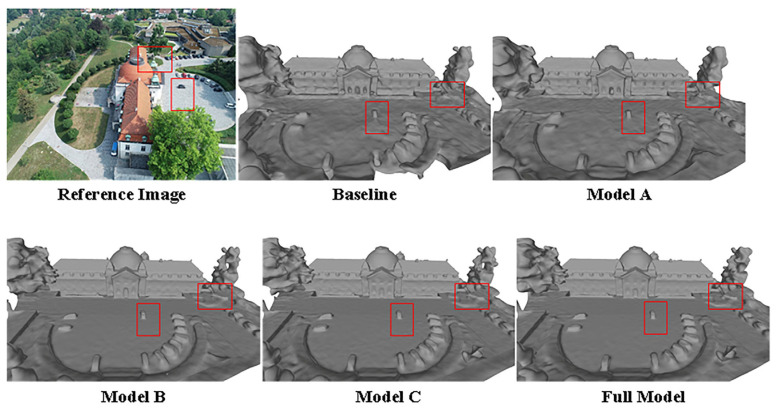
An illustration for the ablation study.

For the ablation experiments, we utilized the dome church data from the BlendedMVS dataset, with NeuS serving as the baseline. We sequentially incorporated additional modules, and qualitative surface reconstruction results are illustrated in Figure 10. In the baseline, the geometric structure is distorted, the surface exhibits significant noise, and the reconstruction area is incomplete. Model A achieves coverage of the entire area but still contends with substantial surface noise. Model B not only completes the reconstruction of the entire area but also notably enhances the geometric structure. Model C further refines the geometric structure, with errors comparable to Model B. In contrast, the Full model demonstrates outstanding results by accurately reconstructing geometric structures and reducing surface noise. Results of the ablation study are reported in Table 6.

In summary, the appearance embedding module appears to be more inclined toward capturing scene details, geometric constraints contribute to improving the quality of geometric reconstruction to a certain extent, and weight constraints effectively enhance model accuracy.

## 6. Conclusions

We introduce Res-NeuS, an approach for photogrammetric neural surface reconstruction. Res-NeuS achieves high surface reconstruction accuracy for remote sensing scenes. Res-NeuS unlocks the capability of ResNet-50 to extract deep image information for neural surface reconstruction modeled as SDF. We show that Res-NeuS proficiently reconstructs intricate scene structures in both object-centric captures and extensive, large-scale scenes with exceptional fidelity. This effectiveness allows for detailed large-scale reconstructions from multi-view images. To mitigate stochastics and ensure the sufficient sampling of details, we use long training iterations. It is our future work to build a faster framework with large-scale processing capability. The network of Res-NeuS can be used directly for image retrieval and image classification in the future.

## 7. Patents

The work reported in this manuscript has been filed for a patent with the following title: Multi-View 3D Reconstruction Method Based on Deep Residuals and Neural Implicit Surface Learning. The application number of this patent is 2023118570901.

## Figures and Tables

**Table 1:** Summary of multi-view 3D reconstruction methods.

Multi-View 3D Reconstruction			Merit	Limitation
Explicit representation	Point clouds		Handles arbitrary topologies	Discrete, limited resolution
	Depth map			
	Mesh			
Implicit representation	Signed Distance function		Continuity; high spatial resolution	Three-dimensional structures are obtained by rendering
	Occupancy field			
	Neural networks encode implicit functions	Surface rendering	Continuity; high spatial resolution	With mask supervision
		Volume rendering	Continuity, high spatial resolution; without mask supervision	Large-scale scene geometry blurring, color, and weight bias

**Table 2:** Architectures for ImageNet.

Layer Name	Output Size	ResNet-50 (50-Layer)
Conv1	112 × 112	7 × 7, 64, stride 2
Conv2_x	56 × 56	3 × 3 max pool, stride 2
		$\left[\begin{array}{cc} 1\times 1, & 64\\ 3\times 3, & 64\\ 1\times 1, & 256 \end{array}\right]\times 3$
Conv3_x	28 × 28	$\left[\begin{array}{cc} 1\times 1, & 128\\ 3\times 3, & 128\\ 1\times 1, & 512 \end{array}\right]\times 4$
Conv4_x	14 × 14	$\left[\begin{array}{cc} 1\times 1, & 256\\ 3\times 3, & 256\\ 1\times 1, & 1024 \end{array}\right]\times 6$
Conv5_x	7 × 7	$\left[\begin{array}{cc} 1\times 1, & 512\\ 3\times 3, & 512\\ 1\times 1, & 2048 \end{array}\right]\times 3$
	1 × 1	Average pool, 256-d fc, SoftMax

**Table 3:** Quantitative results for surface reconstruction of the sculpture on BlendedMVS.

Method	Distance		
	Acc↓	Comp↓	Overall↓
NeuS	0.215	0.00128	0.108
Appearance Embedding	0.176	0.00108	0.089

**Table 4:** Quantitative results for the neural rendering of the sculpture on BlendedMVS.

Method	PSNR↑	SSIM↑	LPIPS↓
NeuS	28.24	0.909	0.077
Appearance Embedding	33.99	0.982	0.039

**Table 5:** Quantitative results for BlendedMVS scenes. The evaluation metric for reconstruction completeness (Comp) is being displayed.

Method	NeuS	COLMAP	Ours
Scene			
scene 3	0.125	0.119	0.117
scene 4	0.00128	0.0425	0.000810
scene 5	0.297	1.75	0.0877
scene 6	1.18	**0.165**	**0.581**
scene 7	3.475	**0.385**	**0.469**
scene 8	0.285	0.242	0.237
scene 9	2.11	1.89	1.84
scene 10	0.425	0.87	0.421
scene 11	1.32	0.692	0.510
scene 12	0.990	0.921	0.102
scene 13	0.121	0.234	0.061
scene 14	0.540	0.370	0.319
scene 15	1.306	0.454	0.311
scene 16	0.213	0.232	0.120
mean	0.884	0.597	0.369

**Table 6:** Quantitative results from ablation models.

Method	Appearance	Weight Constraints	Geometric Constraints	Comp↓
Baseline				0.2134
Model-A	✓	✓		0.1725
Model-B		✓	✓	0.1289
Model-C	✓		✓	0.1275
Ours	✓	✓	✓	0.1203

**Figure A1:**
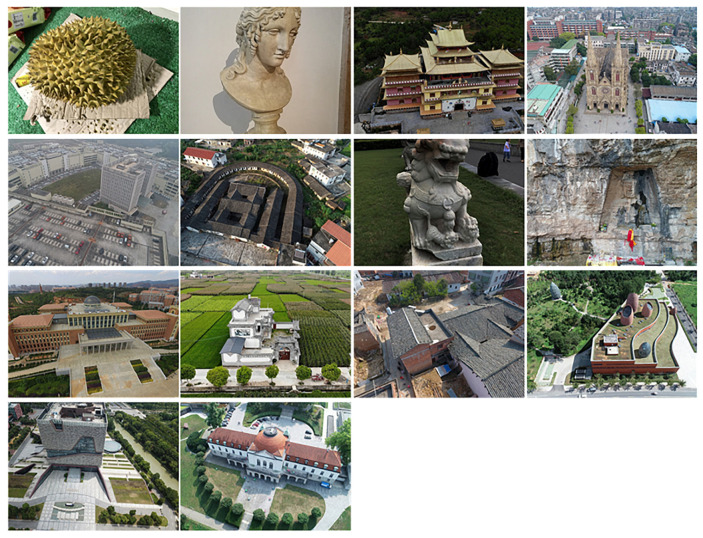
The scenes in the Blended MVS dataset that we used in our work, from left to right and from top to bottom, are scene 3 to scene 14, respectively.

## Data Availability

Data are contained within the article.
